# The psychological effects of research participation on people with dementia: findings from a German exploratory interview study

**DOI:** 10.3389/frdem.2024.1421541

**Published:** 2024-08-07

**Authors:** Katja Seidel, Claudia Winiarski, Jochen René Thyrian, Julia Haberstroh

**Affiliations:** ^1^Department of Psychology, Psychological Aging Research, Faculty V: School of Life Sciences, University of Siegen, Siegen, Germany; ^2^German Center for Neurodegenerative Diseases (DZNE), Greifswald, Germany; ^3^Institute for Community Medicine, University of Greifswald, Greifswald, Germany

**Keywords:** patient participation, participatory research, dementia, psychology, qualitative research, patient engagement, stakeholder engagement, patient and public involvement

## Abstract

The German National Dementia Strategy aims to engage people with dementia in research projects. However, the effects of such research participation on experience and behavior have been insufficiently explored. This study aimed to investigate the psychological effect of research participation on people living with dementia. In a qualitative, exploratory approach, guideline-based interviews were conducted with four persons with dementia who had served as co-researchers on an advisory board in a health services research study for 8 months at that time. The analysis revealed predominantly positive effects of research participation at all levels of experience and behavior. Most effects were reported by the co-researchers on a cognitive level. Both the perception of being competent and of making a positive contribution to oneself and/or others are key effects of research participation. The main effects on an emotional level were joy and wellbeing and on a behavioral level were positive social contacts and social communication. Sadness and insecurity represent the sole negative effects. Nuanced focal points of effects among the individual interviews were found. The results align with existing research highlighting the positive effects of participation on people with dementia. Through advancing an interdisciplinary perspective on their research involvement, we advocate for heightened attention to this topic within the realm of psychology.

## 1 Introduction

Although the *UN Convention on the Rights of Persons with Disabilities* guarantees their right to equal participation and codetermination in political and social decision-making processes, people with dementia, as well as people with other forms of disability, still experience exclusion from decision-making, especially on issues that affect their own lives (Hirschberg, 2010). Participation—in the broader sense understood as access to and involvement in activities, decisions and processes that affect the shaping of social conditions (Arbeitskreis Kritische Gerontologie der DGGG, 2016)—is a human right and a political and civic mandate (Hirschberg, 2010). Persons living with dementia are too often denied the ability to make self-determined decisions about their medical treatment (Wied et al., 2019, 2021) or are excluded from considerations about their own care without the opportunity to address this exclusion (Thraves, 2015). Furthermore, despite growing interest, they are still excluded from many areas of research and are rarely given the opportunity to participate in projects as co-researchers (Rivett, 2017). However, when it comes to health research, there is a scientific approach in the form of participatory health research (PHR) (Wright et al., 2016, 2021) that aims to maximize participation for people whose areas of life or health problems are the subject of research. PHR specifically regards target groups as co-researchers who need to be involved in research processes as equal partners to generate relevant knowledge in the co-production process (Wright et al., 2016, 2021). This should lead to greater health equity (Wright et al., 2016, 2021). This is also reflected in the German National Dementia Strategy (NDS), which aims to “improve health services and the quality of life of people with dementia in line with their needs and requirements” through a variety of activities (p. 132) (BMFSFJ, 2020, p. 132). And Vinay and Biller-Andorno (2023) showed that most of the National Dementia Strategies they included in their evaluation contain patient empowerment as a key ethical aspect. An important field of action within the NDS is to open research in terms of content and methodology by involving persons with dementia in participatory research projects (BMFSFJ, 2020).

Consistent with academic perspectives and scientific evidence, the involvement of people with lived experience is associated with a greater likelihood of positive research outcomes, increased likelihood of applicability and sustainable implementation of healthcare projects (Di Lorito et al., 2017; Bethell et al., 2018; Gregory et al., 2018; Burton et al., 2019; Clar and Wright, 2020; Dening et al., 2020; Schlechter et al., 2021; Brooke, 2019; Tanner, 2012). Alongside the ethical and moral obligation and the instrumental benefit of involving those who are affected in research projects that concern their lives, another perspective on participation can also be adopted.

From a psychological perspective, participation is not only a means of enriching and improving research results through the personal experience of people with dementia. Rather, it can also positively influence the experience and behavior of the people involved. Qualitative studies have reported that they experience positive social relationships as part of their involvement in research projects; feel pride in meaningful activities; report intellectual stimulation, joy, feelings of appreciation, and meaning in life; feel dignity; and perceive their own lives as meaningful despite their illness (Tanner, 2012; Ashcroft et al., 2016; Brooke, 2019; Dening et al., 2020). Participation is also already being used specifically as a means of promoting recovery due to its beneficial effects (Ashcroft et al., 2016). Based on this, it could be assumed that by influencing a person's mental processes and states in a beneficial way, participation can be understood as a (psychological) intervention, defined as “the act of interfering with the outcome or course especially of a condition or process (as to prevent harm or improve functioning)” (Merriam-Webster, (n.d.b)). However, these findings usually appear to be embedded in other questions and tend to be more of a narrative nature. Furthermore, these publications often have methodological shortcomings, particularly regarding the description of the type and extent of participation and are rarely published in renowned journals (Bethell et al., 2018).

There has been an increase in the literature on participatory methods in the field of dementia research, especially since 2019 (Reyes et al., 2023), and a general increase in research activities in the field of participatory research. In the field of PHR, there are a few recent framework models that attempt to structure the potential impact dimensions of participation (Staley, 2015; Banks et al., 2017; Kongats et al., 2018). Any form of research participation can be viewed as a complex intervention with various dimensions of impact, whereby the effects themselves are multifactorial, i.e., influenced, for example, by the project objectives, the commitment of the participants, the group dynamics, and the communication style (Weidekamp-Maicher, 2021). Nevertheless, there is a lack of reliable findings on the question of the psychological effects in terms of benefits for persons with dementia (Ashcroft et al., 2016; Bethell et al., 2018; Brooke, 2019). To the best of our knowledge, there are no empirical studies in which concrete psychological constructs have been specifically derived or systematically determined.

Therefore, in the current research we focus on the effects on the subjective experience of people with dementia. The aim is to gain a better understanding of the psychological effects and potential benefits of research participation for them. Specifically, the effects of participation will be investigated from a psychological background using an exploratory approach. In the context of the present work, it seems crucial to emphasize that people with dementia are a particularly vulnerable group in the context of research activities. Cognitive impairments, above all those affecting memory, the planning and control of actions, a limited ability to abstract and the loss of communication skills can cause methodological problems when conducting projects and research with people with dementia (Slegers et al., 2015; Di Lorito et al., 2017). People with cognitive impairments may perceive their world and share their experiences differently, which can present challenges when carrying out projects together with them (Slegers et al., 2015). Another limitation for their participation is the concern about their ability to give informed consent to research (Swaffer, 2016). These challenges concern not only the research process itself, but also the resulting research findings, which may be affected. When investigating our research question, we try to take these challenges into account.

## 2 Materials and methods

### 2.1 Study design

The analysis of the psychological effects of participation follows a qualitative, exploratory design using semi-structured, guideline-supported interviews. The reporting of the methods applied in this study is aligned with the Consolidated Criteria for Reporting Qualitative Research (COREQ) (Tong et al., 2007) and the Standards for Reporting Qualitative Research (SRQR) (O'Brien et al., 2014).

### 2.2 The participatory research project as a framework

In cooperation with a local Alzheimer Association (AlzA), two advisory boards (persons with dementia and relatives of persons with dementia) were established in 2021 as part of the *Participatory Pilot Study DelpHi-SW* (Dementia: lifeworld-oriented and person-centered support in Siegen-Wittgenstein). DelpHi-SW tested a structured participatory approach to adapt the evidence-based complex dementia care management intervention (DeCM) (Thyrian et al., 2017) to an exemplary regional setting in Germany (Seidel et al., 2022) and prepared it for a subsequent implementation study (Purwins et al., 2023). The advisory board members (ABM) advised on and helped shape the regional and cross-sectoral adaptation and implementation of the DeCM. Their responsibilities included setting topics for DeCM, revising information materials and survey instruments, and discussing issues relating to the concrete implementation of the study. Feedback was reported to other stakeholders and the project team and was incorporated into the DeCM study. The advisory board meetings have been held once a month since July 2021, each lasting 1.5 h They were held in a more familiar setting, accompanied by two academic researchers (KS, female psychologist) and moderated by two experienced AlzA moderators. In the course of dementia, there is an increasing loss of cognitive performance. Alzheimer's disease in particular leads to progressive losses in communicative abilities along the four communication steps of *Presentation, Attention, Comprehension*, and *Remembering*, as described in more detail in the *TANDEM communication model* by Haberstroh et al. (2011). Disease-related language limitations therefore represent a potential barrier when working with persons with dementia as research partners. Strategies are already available, such as the evidence-based training program *TANDEM* by Haberstroh and Pantel (2011). The following communicative strategies, amongst others, appeared to be relevant for the work within the advisory board: linking to old memories and life themes, linking to universal experiences, “What for?” questions, biography work, helping to find the thread again, attentive posture, responding to unfamiliar words in a non-concrete way (Haberstroh and Pantel, 2011).

### 2.3 Participants

The exploratory interview study was conducted with *N* = 4 participants (two females) who were between 45 and 80 years old and had a mild degree of dementia of various types with only slightly pronounced psychological and behavioral symptoms. Prior to the collaboration with the ABM and before the interview study, we made the decision not to assess the degree of dementia development. We believe that such an approach would not have been appropriate because it would have been associated with a deficit-oriented attitude toward our co-researchers, would have reminded them more of a patient role and would have made anonymization even more difficult. The psychological and behavioral symptoms became evident during the meetings, e.g., in the form of slight memory loss, difficulty finding the right words for something and/or following complex conversations, attentional fluctuations, or mild mood swings (sadness, impatience). At this point, all interviewees had been ABM for 8 months. All interviewees had sufficient hearing and vision. Interview participation was voluntary, and no financial or other compensation was granted. Ethical review and approval were obtained from the Council for Research Ethics at the University of Siegen (ER_27/2021).

### 2.4 Materials

Due to the lack of systematic research on the psychological impact of research participation on persons with dementia, no established questionnaire could be used. We therefore developed an interview guide (see Supplementary Table 1) using the so-called *SPSS method* (German language abbreviation for collect, check, sort, subsume) (Helfferich, 2011). First, as many questions as possible on the participatory effect of the advisory board's activities were collected. These questions were then critically checked by the academic researchers to determine whether, for example, they stimulate narration, touch on the relevance systems of the co-researchers and do not ask for facts (Helfferich, 2011). The remaining questions were then bundled and sorted by content. The interview partners were not involved in the development of the interview guidelines.

### 2.5 Data collection

The four individual and audio recorded interviews took place in March 2022 in the home setting of the four ABMs without the presence of third parties. The interviews lasted 47, 30, 54, and 10 min and were conducted by the academic researcher (KS). The interviewees were informed orally and in writing about the content, aim, potential risks, and audio recording of the interview study. To ensure informed consent, relevant material was adapted regarding dementia-sensitive language and based on documents already drafted by the advisory board members. The consent of the interviewees was continuously checked throughout the entire interview process so that the interviews could be terminated in the event of discomfort, stress, or unwillingness. In two interviews, the academic researcher and the interviewee jointly decided to end the interview due to increasing emotional arousal. Both interviews were included in the analysis, as the main topics had already been addressed in both interviews. Both persons accepted the offer of a consecutive stabilizing conversation. Depending on the particular needs of the interviewees, they were able to express and/or verbalize their emotions in this conversation. With reference to statements already made, the focus was then directed to existing resources or further services. After the interviews, postscripts with additional information on the interview situations were created.

### 2.6 Data preparation and analysis

To capture speech delays, word-finding inhibitions, and simultaneous speech, all interviews were transcribed (CW, psychologist) according to the extended content-semantic transcription system (Dresing and Pehl, 2015). The transcripts were checked against the audio recordings by the interviewer and supplemented with para- and non-verbal aspects. In the end, a total of 19,095 words were generated. The transcripts were then anonymized according to *Bochumer Anonymisierungsmodell* (*Bochum anonymization model*; Richter et al., 2021) via a combination of factual and absolute anonymization.

Qualitative data were analyzed according to structuring content analysis by Kuckartz and Rädiker (2022) using the software MAXQDA.^1^ For this purpose, after (1) initiating text work, both researchers independently and inductively (2) developed thematic main categories, (3) coded the entire material accordingly, (4) summarized the text sections with the same coding, (5) inductively formed subcategories, (6) coded the entire material again with the main and subcategories, and (7) analyzed the data. This involved a category-based analysis along the lines of the main categories, an examination of correlations between the interviews and particularities at the individual case level. Divergent coding was critically discussed, and final coding was conducted by consensus.

## 3 Results

Overall, 23 main categories were created from 246 text units, whereby text passages were also assigned to several categories. These main categories can be classified along three dimensions: *emotional level, cognitive level*, and *behavioral level*. With 104 text units (42%), most of the codes are assigned to eight main categories of the cognitive level, 81 text units (33%) to nine main categories of the emotional level, and 61 text units (25%) to six main categories of the behavioral level. Table 1 presents the overall results of the coding process.

**Table 1:** Overall results of the coding process: main categories and their subcategories sorted by dimensions.

Main category^*^	***n***	%^**^
**Emotional level**	**81**	
Joy	20	25
Anticipation of the advisory board	4	
Through participation in the form of the advisory board	16	
Wellbeing	17	21
Sense of belonging and integration	12	15
Pride	12	15
Emotional relief	7	9
Feeling supported	6	7
Security	3	4
Sadness	2	2
Insecurity	2	2
**Cognitive level**	**104**	
Competence experience	24	23
Making a positive contribution	19	18
For oneself	4	
For others	15	
Satisfaction with advisory board activity	15	14
Cognitive stimulation	14	13
Reflection on one's own needs	13	13
Reflection on one's own dementia disease	11	11
Dementia as part of the self	5	
Perception of deficits within the advisory board	6	
Confidence	5	5
Curiosity	3	3
**Behavioral level**	**61**	
Social communication	26	43
Within the advisory board	20	
About the advisory board	6	
Social contacts	12	20
Contributing resources	8	13
Other activities	6	10
Being authentic	6	10
Social participation	3	5

*N* = 246 text units.

^*^Subcategories are right aligned.

^**^The proportion of main categories and subcategories within the respective dimension.

While the three most frequently mentioned main categories of the dimensions of emotion (*Joy, Wellbeing, Sense of belonging and integration*) and cognition (*Competence experience, Making a positive contribution, Satisfaction with advisory board activity*) can be found in all interviews, the distribution and focus of the other categories differed across the four interviews. Therefore, the following results, structured by dimension, focus on the three aspects of experience and behavior that were most frequently described by the respondents in connection with their participation as an ABM. All other categories with sample statements are shown in Supplementary Table 2. Additionally, specifics at the individual case level are also reported. All quotations are presented linguistically in their original form and capitalization is used to make special linguistic emphases visible.

### 3.1 Emotional level

#### 3.1.1 Joy

This category refers to a feeling of pleasure and happiness that, in contrast to wellbeing, does not describe a global feeling but rather a feeling that is linked to concrete events (Wirtz, n.d.). Our results show that joy can refer to the anticipation of the advisory board and to the enjoyment caused by participation in the advisory board itself. The initial anticipation was already evident with the request to join the advisory board:

“You know what, right away. I didn't even think about it, right away yes” (Interview 2, pos. 61–64).

This joy remained even after the start of the project. When asked how they felt when they knew it was the day of the advisory board meeting, the interviewees stated:

“Well, actually I'm always looking forward to it like hell” (Interview 2, pos. 136–139).

“I always find it, am always somewhere actually, when these appointments are, um yes, in such a positive tension” (Interview 3, pos. 31–33).

Joy is also reported in connection with participation in the advisory board itself. The reasons for this are manifold. First, the work itself is enjoyable.

“Therefore, it was just the self-help group, but then I'm really proud, say [name of partner], ‘I have worked hard again!”' (Interview 2, pos. 205-207).

“Well, I, I, you see, I am coming out of my shell…. That makes me happy.” (Interview 1, pos. 648–651).

Joint communication in advisory board meetings is also a pleasure. For example, one interviewee “simply enjoys being able to talk to open people” (Interview 1, pos. 346–347). Another emphasizes this aspect:

“And that was a very short, but VERY intense statement, where I thought: great, amazing, good.” (Interview 3, pos. 298–303).

After all, it is also their own advisory board work that gives them pleasure. When asked how it felt to know that their own suggestions would be considered in the study project, one advisory board member replied:

“Very, very, very, very, very, very, very, VERY nice. Yes.” (Interview 2, pos. 310–313).

#### 3.1.2 Wellbeing

This category includes all text units in which the interviewees describe the perception of being happy and generally satisfied, frequently experiencing positive and rarely negative experiences and feelings (Eid, 2021). Most of the interview statements encoded refer to the advisory board in general. Descriptions such as “incredibly pleasant” (Interview 1, position 634); “pleasant atmosphere” (Interview 3, position 41–42); “such a very um very pleasant atmosphere” (Interview 3, pos. 41–42); “how well one is feeling” (Interview 2, pos. 658); or “I truly LIKE going there” (Interview 1, pos. 250) were often used. One interviewee described it more vividly:

“Almost as if in God's hands.” (Interview 1, pos. 645).

According to a smaller number of codes, *Wellbeing* is explicitly linked to the personal commitment of the academic researchers, who are perceived, for example, as “nice and kind” (Interview 1, pos. 684).

#### 3.1.3 Sense of belonging and integration

This category refers to the feeling of belonging and being integrated into a group and/or topic on an equal level (Merriam-Webster (ed.), (n.d.a)). The co-researching ABM felt that they belong, e.g., to “like-minded people” (Interview 4, pos. 25), to “people affected [by dementia]” (Interview 3, pos. 36) to “other colleagues” (Interview 1, pos. 446), or to people with whom “you can talk about something like that [dementia]” (Interview 1, pos. 207).

“But um, it was nice, they were all people with dementia…. And talking to them and such. I thought it was nice… With like-minded people like that” (Interview 4, pos. 19–25).

The perception of *belonging and integration* also occurs when the ABM perceive themselves as actively involved in the board activities. Thus, praised the “openness” (Interview 1, pos. 148) to collaboration, saying that one could “suddenly participate on a completely different level” (Interview 1, pos. 525–526). When asked how to recognize good involvement, one interviewee replied:

“You know what, you all ask us. Everyone can add their two cents” (Interview 2, pos. 271–272).

### 3.2 Cognitive level

#### 3.2.1 Competence experience

Perceiving oneself as competent is the most frequently assigned main category on a cognitive level. The co-researchers felt that they were able to successfully fulfill the tasks and performance requirements of the advisory board through their own actions (Wirtz, 2021). This impact of participation can be seen in various aspects. First, in the realization that they are competent, -for example, “in an area that I can still follow relatively well” (Interview 3, pos. 182). After the meetings, one advisory board member “had the feeling that I had done something good for the advisory board and for myself” (Interview 2, pos. 147–148). Second, the perception of being asked for advice also signaled an attribution of competence:

“So, we are not stupid, we are still quite alive” (Interview 1, pos. 668–670).

Third, all four co-researchers also explicitly confirmed that they did not feel overwhelmed by their participation in the advisory board. One person reported:

“Yes, I feel a bit challenged. Not that it's too much for me” (Interview 1, pos. 517–518).

Challenged without being overwhelmed is also how this advisory board member sees it:

“I noticed that when we did something. I did notice that, right…. Yes, always a bit. But you know, I was [at work] before. I was physically exhausted and mentally exhausted. That was ideal” (Interview 2, pos. 483–489).

Regarding their advisory board activities, the co-researchers not only perceived themselves as competent in real-life situations but also saw themselves as capable in the future:

“Well, I think I'm still someone who can contribute good ideas and has the fantasy to do so. Or simply, um, yes, all these stories that you have somehow experienced” (Interview 3, pos. 433).

When asked whether they would have thought that participation would be possible despite the health restrictions, one person resolutely answered “Yes, I think so” (Interview 4, pos. 190). The four co-researchers not only felt capable, but also, in some cases, attributed a value to their own actions, as explained in the following category.

#### 3.2.2 Making a positive contribution

According to the co-researcher's definition, this main category is based on the realization that their own advisory board activities contribute to a result that they personally perceive as valuable. A further distinction can be made between the positive contributions for oneself and for other people or circumstances (as subcategories). The former can be seen in statements such as:

“Yes, but that also has something that helps me to say where I am now on my path” (Interview 3, pos. 249).

“And it also helps me at the same time” (Interview 1, pos. 293).

Being a co-researcher also provides access to relevant information and “that is also important for us, not just for you” (Interview 2, pos. 30–31).

However, most of the coded statements relate to the positive effect of one's own activity on others.

“Yes, um I have the feeling that despite everything I can somehow still contribute and somehow still pass on certain things” (Interview 3, pos. 612–613).

The positive aspects can also be directed toward the other co-researchers, for example, when their own strengths are brought into the advisory board:

“Yes, that means a bit, I'm doing well. And, I can see from the applause that it does the others good too” (Interview 1, pos. 329–331).

#### 3.2.3 Satisfaction with advisory board activity

This category refers to all text segments in which the co-researchers' perception is expressed that the expected and achieved personal goals within the framework of the advisory board activity coincide (Zufriedenheit [satisfaction], 2000). When asked whether their own expectations had been met, one person replied:

“And HOW!” (Interview 2, pos. 678)

“In any case, in EVERY case” (Interview 2, pos. 67).

In some cases, satisfaction is explicitly linked to the framework conditions perceived as harmonious, for example, regarding the frequency of the advisory board meetings (Interview 2, pos. 693–694; Interview 3, pos. 28–31) or the interpersonal atmosphere (Interview 1, pos. 546–548). Indirectly, the level of satisfaction of the ABM with their participation can be deduced if they recommend participation to other people with disabilities.

“Yes, I can only say what I thought was right for me. And, please, help yourselves.” (Interview 1, pos. 354–355)

“Well, everything here was super great. The people should come.” (Interview 2, pos. 655–656).

### 3.3 Behavioral level

#### 3.3.1 Social communication

At the behavioral level, the effects of participation are predominantly evident in the category of social communication, understood as the mutual exchange of information about thoughts and feelings (Bierhoff, n.d.). The results suggest a further differentiation between communication outside the advisory board about the advisory board and communication within the advisory board on general and disease-related topics (as subcategories). In the context of disease-related topics, the central value of participation in the advisory board becomes apparent.

“You can talk about it….How it goes for everyone and what they do” (Interview 4, pos. 42–44).

“I always think it's good that all these people come together. That we can talk to each other. Because one person does it one way and another person does it differently.” (Interview 4, pos. 75–78).

One would “simply enjoy being able to talk to open people.” (Interview 1, pos. 346–347), and it would be “so relaxed and nice about such an UNpleasant … topic” (Interview 1, pos. 306).

The individual's responsibility as an advisory board member is formulated as follows:

“That I also say um difficult things… and that helps me, of course, that I can get it off my chest.” (Interview 1, pos. 49–55).

The open, inviting culture of discussion is emphasized by the statement that the advisory board is about “mental work and, um, telling stories” (Interview 2, pos. 368) and that everyone can “add their two cents” (Interview 2, pos. 271–272). In the context of non-illness-related communication, the possibility of so-called wellbeing rounds is appreciated.

Outside of the advisory board, the main contacts for discussion of advisory board topics are not only partners and family but also work colleagues. In addition, the advisory board also becomes a topic among friends:

“A lot of people know about me, um, that I have Alzheimer disease somewhere… Um, and with individual friends… where it goes a bit further, um, I also gave them details.” (Interview 3, pos. 544–548).

#### 3.3.2 Social contacts

Our results show that the advisory board offers all co-researchers the opportunity to make positive social contacts. On the one hand, this refers to the academic researchers.

“I feel comfortable with you… You are nice and kind.” (Interview 1, pos. 380–382).

However, above all, the advisory board is described, for example, as a “nice group” (Interview 3, pos. 51).

“They were all people with dementia…. And, talking to them and all that. I thought it was nice… We were just among ourselves.” (Interview 4, pos. 19–59).

#### 3.3.3 Contributing resources

On the advisory board, the co-researchers were able to contribute their own resources, for example, personal topics, abilities, and skills. This concerns, for example, “All these stories that you have somehow experienced, um, as a [profession].” (Interview 3, pos. 433).

“Well, I think I'm still someone who can come up with good ideas somewhere, and has the imagination to do so.” (Interview 3, pos. 433).

### 3.4 Additional findings

There are distinct subcategories for the main categories of *Joy, Reflection on one's own dementia disease*, and *Social communication*, which do not overlap in the coding. This is not the case for the perception of *Making a positive contribution*. Here, the co-researchers think simultaneously about making a positive contribution both for themselves and for others. For example, when speaking of a “…win–win situation. I would like to help the other people and help myself too.” (Interview 2, pos. 26–27).

Furthermore, there are connections between the different main and sub-categories. A text segment can address several thematic aspects, which is why several main and sub-categories can overlap or nest within one another. Such overlap can be observed particularly frequently in the behavioral category of S*ocial communication within the advisory board*. This applies, for example, to the combination of *Social communication* and *Being authentic*. Furthermore, *Social communication* is often flanked by emotional experience components. Several text segments on internal advisory board communication are also labeled with the main code *Emotional relief*. In this way, members of the advisory board can “talk things out” (Interview 4, pos. 42) and “get rid of stressful thoughts” (Interview 1, pos. 54, pos. 137–138). A *Sense of belonging and integration* is also often described when the ABM talk to each other. This generally applies when the co-researchers are among “like-minded people” (Interview 4, pos. 22–27) with whom they can talk about their own dementia. Both, *Social communication within the advisory board* and the *Sense of belonging and integration* are closely linked to the behavioral category of positive *Social contacts*. For the latter, the results often show a connection with the emotional category of *Wellbeing*. This applies both to contact between co-researchers and academic researchers and to contact among the ABM. The cognitive category *Competence experience* was also frequently coded together with other categories, such as the category *Making a positive contribution to others*. In the corresponding text segments, the co-researchers reported, for example, that they “did something good” for the advisory board (Interview 2, pos. 147–148) or “helped to help others” (Interview 1, pos. 732–733). On an emotional level, this perception of expertise is often accompanied by *Pride*. For example, when the co-researchers “worked hard again” (Interview 2, pos. 207) or participated “on a completely different level” (Interview 1, pos. 525–526) in the board meetings.

Regarding the psychological effects of participation as an ABM, different central themes can be identified for each co-researcher. In interview 4, statements coded to the three categories *Social contacts, Social communication within the advisory board*, and *Sense of belonging and integration* are mentioned particularly often and are interwoven with each other. This combination of categories accounts for more than half of the codes in this interview. For this co-researcher, the advisory board primarily offers the opportunity to *communicate with other people about dementia*, to make positive *social contacts* and to behave *authentically*. On an emotional level, this person feels disproportionately comfortable, particularly *relieved* and *supported*. Interview 2 focused on cognitive aspects, and the advisory board was equated with *cognitive stimulation* with striking frequency. On an emotional level, this is accompanied by great j*oy* and *pride*. In interview 3, the differentiated *reflection on needs and* d*ementia* was striking. Only this co-researcher talks about the negatively connoted perception of deficits in the context of the board's work and describes negative feelings of *insecurity* and *sadness*. On the other hand, this person feels *confident* and often *competent*. This experience of competence is linked to the two categories of *contributing resources* and the perception of *making a positive contribution to others*. On a personal level, participation in the advisory board had such predominantly positive effects that the person decided to be involved in other working groups as well.

## 4 Discussion

As one of the first studies from an explicitly psychological perspective, this project investigated the psychological effects of research participation on persons with dementia. We found various psychological effects along the three dimensions of emotion, cognition, and behavior, with a focus on the cognitive level across all interviews. As expected, and in line with the literature, the present study also shows that the impact of advisory board activity on co-researchers is of significant importance (Staley, 2015; Swarbrick et al., 2019). For reasons of clarity, we discuss the results according to the dimensions found.

### 4.1 Cognitive effects of research participation

On a cognitive level, it is noticeable that the co-researching ABM often perceive themselves as competent and are able to verbalize this. This finding is consistent with previous literature and can be found both in general studies about research partnerships (Hoekstra et al., 2020) as well as in studies involving people with dementia (Clare et al., 2008; Tanner, 2012; Littlechild et al., 2015; McConnell et al., 2019). In the context of research participation, a minimum level of skill, such as in spatial orientation, attention, and language, is required (van Baalen et al., 2011). In terms of language skills, mildly affected persons are more likely to understand rather simple verbal messages, and memory and word finding may already be impaired, but grammar and attention are still largely intact (Kuemmel et al., 2014). With suitable methods, people with dementia with early-onset impairments in particular can therefore formulate and represent their thoughts, feelings, and interests themselves (Aggarwal et al., 2003; Wißmann, 2021).

The cognitively stimulating character of the advisory board meetings is perceived positively by the co-researchers and, in their view, distinguishes the advisory board from the meetings of the self-help group. Ashcroft and colleagues (Ashcroft et al., 2016) were previously able to identify intellectual stimulation as a positive effect of participatory involvement. This is relevant because wide-ranging cognitive stimulation, which includes sensory experiences, positive memories, communication, and social contact, can help to preserve the remaining cognitive resources of persons living with dementia (Ivemeyer and Zerfaß, 2006). It is also noticeable that the ABMs not only perceive themselves as competent but also attribute positive value to their actions. The perception of making a positive, meaningful contribution to oneself and/or others in the context of research participation has already been extensively document-ed in the literature (Fudge et al., 2007; Steeman et al., 2007; Littlechild et al., 2015; Ashcroft et al., 2016; Waite et al., 2019). Some of the statements made also describe a *give and take* in the context of their advisory board activities. If those affected give back the support they receive through their own contributions, this can in turn have a positive effect on their subjective wellbeing—a fact that could also be expanded as part of targeted interventions (Godde et al., 2016).

Our results also show the emancipatory potential of participatory projects discussed in the literature (Clare et al., 2008; Arbeitskreis Kritische Gerontologie der DGGG, 2016; McConnell et al., 2019). In this way, the co-researchers continue to experience themselves as effective by contributing their individual competences and strengths and experience themselves as competent in the sense of self-efficacy and making a positive contribution to themselves and others. This is a relevant aspect, as the personal resources of the co-researching persons are understood as protective factors that can support coping with their disease and improve their quality of life and wellbeing (Arbeitskreis Kritische Gerontologie der DGGG, 2016; Gruber, 2020).

### 4.2 Behavioral effects of research participation

A very significant, positive effect of the advisory board's activities can be seen at the behavioral level in the form of social communication and positive social contacts. This finding is also not surprising, as a positively perceived expansion of the social network has already been documented (Fudge et al., 2007; Litherland et al., 2018; Hoekstra et al., 2020). As our findings show, the co-researchers even feel encouraged to be able to behave authentically among people with the same condition. Participating in interesting projects together with others also prevents from withdrawing at home.

### 4.3 Emotional effects of research participation

On an emotional level, the advisory board represents joy and wellbeing for the co-researchers with almost half of all coding in the interviews falling into these two categories. How central a shared joyful experience is for people with disabilities is shown by the fact that fun is considered one of the key therapeutic principles of *cognitive stimulation therapy* (Aguirre et al., 2018). Our results also confirm findings showing that people living with dementia have a great need for appreciation and recognition (Niebuhr, 2010). This relates to biographical and life experiences, which serve as personal resources for the advisory board, as well as participation in the advisory board itself. The ABMs describe being proud when they receive positive feedback on their participatory involvement, both within and outside the advisory board. Previous studies have shown that co-researchers experience appreciation as part of their research participation (Fudge et al., 2007; Litherland et al., 2018; Hoekstra et al., 2020).

A special feature of research with people with dementia is that co-researchers are inevitably confronted with their dementia as part of their advisory board activities. As described by the ABMs, this stimulates reflection processes that involve an active examination of the condition and the course of their own illness. This has already been considered as an opportunity for individuals to come to terms with their illness (Ashcroft et al., 2016). Providing participatory support for dementia research can even give life with this disease a new, independent value (Clare et al., 2008). However, confrontation with dementia can also have negative, stressful effects on co-researchers. This is particularly true when those affected perceive increasing disease-related limitations and losses (Span et al., 2018). In the interviews, sadness and insecurity were found to be negatively connoted feelings and deficits in the context of the advisory board activity. Interesting, but congruent with previous findings, is the fact that these negative thoughts and feelings do not appear to carry much weight in the overall view of research participation (Ashcroft et al., 2016; Weidekamp-Maicher, 2021). The ABM seem to be able to allow and balance these opposing feelings in the context of their advisory board activities and successfully self-integrate the negative feelings, so that a view of the positively perceived aspects of the advisory board becomes clear again. Other negatively connoted thoughts or feelings, as described in the literature, such as dissatisfaction, the feeling of not being heard and appreciated, or feeling overwhelmed (Ashcroft et al., 2016; Hoekstra et al., 2020) were not addressed by the ABM. On the contrary, the co-researchers reported great satisfaction with the frequency of the meetings, the composition of the advisory board, the working nature of the meetings, and the results of their own advisory board activities.

### 4.4 Additional findings

Our results show a strong connection between social and emotional components. This suggests that the advisory board seems to fulfill basic psychosocial needs. We would like to combine this result with current findings that social and, above all, emotional support are important protective factors for the life expectancy of people living with dementia (Blotenberg et al., 2024). An absence of both appears to be a risk factor for shorter life expectancy, over and above other known clinical factors. Participation can be one way to find social and emotional support. Therefore, our results strengthen the call for greater attention to be given to the psychosocial needs of people with disabilities (Blotenberg et al., 2024).

In the context of research, older people, even those without dementia, are assumed to be uncooperative or uninterested in research (Wanka and Urbaniak, 2023). In contrast, our results show a strong need among ABM to reassure themselves of their remaining competencies by repeatedly addressing their own skills and participative contributions. However, it appears that the application of remaining skills seems to be the central issue. An increase in skills, as described in several studies on patient and public involvement (Fudge et al., 2007; Baldwin et al., 2018; Hoekstra et al., 2020), was not explicitly addressed in the interviews.

Our results show distinct inter-individual differences in the motivation to participate in advisory boards and the psychological impact of research participation. This speaks to the importance of continuing to see persons living with dementia as individuals despite having the same condition and, above all, taking their individual needs and personality into account when working with them as co-researchers.

### 4.5 Strengths and limitations

With the content analysis method according to Kuckartz and Rädiker (2022), a method was chosen that allows *a priori* category formation from empirical data and guidelines as well as inductive, explorative category formation on the material or a combination of both variants. This allowed a previously little investigated research subject to be comprehensively illuminated and described in greater depth. Both the data generation and evaluation followed strict quality criteria. This applies above all to intersubjective traceability, which was ensured above all through detailed procedural documentation, consistent verification by both researchers regarding coding, and the explication and documentation of all research steps. The standardization of procedures, e.g., interview guidelines, transcription, anonymization, and coding rules, increases procedural reliability, i.e., trust in the data and its interpretation. The different perspectives of the two coders are seen as a further strength. While one was an active part of the interviews, the other only knew the interview situation from the audio recordings and postscripts. Critically reflecting on deviating coding and ultimately reaching a consensus on assignments, therefore, meant a very intensive examination of the data material and contributed to internal consistency. The interviews were partly characterized by very long units of meaning, interjections, and digressions regarding the individual characteristics of the interviewees' speech production and comprehension. Communicative validation during the interviews, i.e., summarizing or reflecting the statements to the interviewees, clarified comprehension difficulties and increased the probability that what was said corresponded to what was meant.

Four interviews were not and are not intended to generate results representative of the entire group of persons living with dementia. However, in contrast to the principle of external validity in quantitative research, the focus in qualitative research is on authentic or comprehensive representation (Kruse, 2015). Nevertheless, the characteristics of individual interviewees may have played a greater role in the overall presentation of the results. This is another reason why impact analysis at the individual case level is so important. In line with other literature (Arbeitskreis Kritische Gerontologie der DGGG, 2016), the group of co-researching persons with dementia was also found to have a relatively high level of formal education, socioeconomic status, and no migration background. This is another reason why the results do not aim to generalize and represent a specific group of people. As only people with mild dementia were interviewed, no statement can be made about the experience and behavior of people with more severe dementia. Furthermore, the practical support provided by the personal environment and the AlzA favored the participation of the co-researchers. This indicates that they therefore have considerable social capital (James and Buffel, 2023), which is not the case for the general population of people with dementia. The interviews were conducted by an academic researcher who was known to the co-researchers from the advisory board meetings. Although existing trust and mutual sympathy promote a pleasant and open discussion atmosphere, such an established relationship between speakers could also lead to distortions in response behavior during an interview, e.g., in the sense of social desirability. This applies here in particular because the interviews took place in the middle of the project period, and both parties were interested in a positive evaluation. Unwanted power dynamics between academics and co-researchers must also be considered.

### 4.6 Practical implications

In terms of an interdisciplinary view of participation and, above all, research participation of persons with dementia, we advocate greater consideration of the topic in the realm of psychology. The biopsychosocial model can provide an integrative framework to explain the psychological effects of participation on the co-researchers using established psychological theories. People in the later stages of dementia, those with a migration background and those with insufficient social resources must also be given access to research projects and thus also to the associated positive psychological effects. Based on the findings on the high socioeconomic status of most co-researchers in participatory research projects, this also touches on the ethical issue of perpetuating existing inequalities through participatory research.

In addition, a procedure for dealing with emotionally stressful interview situations with people with dementia should be developed and empirically evaluated.

## 5 Conclusion

The largely positive feedback from the advisory board members shows that people with dementia are very happy to be involved in research efforts and contribute to the knowledge gained as experts of their own lives. Nonetheless, various circumstances must be considered when conducting research with them to enable them to have a positive experience of participation. It is particularly important to create conditions that allow co-researchers to experience the positive effects of their participatory engagement, that they are challenged but not overwhelmed, and that negative emotional reactions to perceived disease-related losses are appropriately addressed. Despite the increasing number of participatory research projects with people with dementia, the impact of research participation on those affected is still not extensively considered (Backhouse et al., 2016; Rivett, 2017; Bethell et al., 2018; Harris et al., 2018). With our study, we would like to contribute to psychology's involvement in the topic.

## Data availability statement

The datasets presented in this article are not readily available because for data protection purposes, no raw data can be made available, as it is highly likely that conclusions could be drawn about individual interviewees from these data. Requests to access the datasets should be directed to katja.seidel@uni-siegen.de.

## Ethics statement

The studies involving humans were approved by Council for Research Ethics at the University of Siegen (ER_27/2021). The studies were conducted in accordance with the local legislation and institutional requirements. The participants provided their written informed consent to participate in this study.

## Author contributions

KS: Conceptualization, Formal analysis, Funding acquisition, Investigation, Methodology, Project administration, Resources, Visualization, Writing – original draft, Writing – review & editing. CW: Formal analysis, Writing – original draft. JT: Funding acquisition, Writing – review & editing. JH: Funding acquisition, Supervision, Writing – review & editing.

## Footnotes

^1^MAXQDA [Computer software] (2022). *VERBI Software*.
