# Supplementary material for: The psychological effects of research participation on people with dementia: findings from a German exploratory interview study
Source: Front Dement. 2024 Aug 7;3:1421541. doi: 10.3389/frdem.2024.1421541 (PMC11335729; doi:10.3389/frdem.2024.1421541)
Supplement: Supplementary file 1 [file Table_1.DOCX]

Supplementary Material

**Table 1.** Structured Interview Guide

| Thematic Aspect | Question |
| --- | --- |
| Entry section | |
| Greetings | A warm welcome to you, <name>. |
| Reason for interview | As we have just discussed, I would like to find out how you experience yourself as a co-researcher in the advisory board. |
| General consent + Audio recording | Thank you for taking the time to talk to me today. Thank you also for allowing me to record the interview. |
| Main section | |
| Understanding of the advisory board | Can you describe in your own words how you understand the advisory board? |
| Expectations when requesting to join the  advisory board | When you were asked whether you would like to join the advisory board what did you think?  What were you expecting from your work as an advisory board member? |
| Satisfaction with advisory board activities | What would you say, have these expectations fulfilled so far?  How satisfied are you with your decision?  Can you provide some examples to illustrate this? |
| Previous engagement | Have you ever been involved in an advisory board, honorary position or similar?  To what extent did this experience help you as an advisory board member? |
| Preparation for the advisory board meetings | We meet once a month for our Advisory Board. How do you prepare for these advisory board meetings?  What experiences have you had with filling out the questionnaires? |
| Thoughts and feelings prior to the advisory board meetings | Can you describe to me what your mood is when you know that it is he day of the advisory board meeting? |
| Social communication within the advisory board | How have you experienced the dialogue within the advisory board so far? Can you provide an example of this? |
| Integration into advisory board work | Do you feel well integrated into the work of the advisory board? How do you recognize this? |
| Skills and strengths | What skills and strengths help you as an advisor?  What helps you to be a good advisory board member?  Can you describe this in more detail? |
| Appreciation through advisory board activity | Do you have the feeling that your opinions and  suggestions help the advisory board move forward? Can you perhaps tell me more about this?  How does it feel when your suggestions are considered within the project? |
| Contributing own topics | You have already done a lot of work on the Advisory board and addressed many topics: How important is it that you also have a space for your own topics?  Can you perhaps describe this in more detail? |
| Thematic Aspect | **Question** |
| Experience of being overwhelmed | Have you ever had the feeling that the work on the advisory board is too strenuous or demanding? How did you notice this?  How did you then deal with such thoughts or feelings? |
| Thoughts and feelings after the advisory board meetings | What is your mood like when you go home after the advisory board?  What goes through your mind? Can you perhaps describe this in more detail? |
| Further activities | Please describe what you do after the advisory board meetings. |
| Social communication outside the advisory board about the advisory board | What experiences have you had with communicating with other people about your involvement in the advisory board?  How do they react when you tell them that you are on the advisory board?  How does that make you feel? |
| Recommendation to other people with dementia | Would you also recommend other people with dementia to get involved in advisory boards?  What would you say to other people with dementia if they are unsure whether they should participate in advisory boards?  Is there anything else to add? |
| Potential interest to continue involvement | Would you like to stay involved in an advisory board?  What would be your motivation? |
| Wishes for the advisory board | If you were to continue to be a member of an advisory board, what wishes would you have for the new advisory board? |
| Regional affiliation | How important is it to you that the advisory board is involved in research activities specifically for your region? |
| Exit section | |
| Open exit | We have already discussed several topics.  Is there anything else you would like to say that is important to you that has not yet been discussed? |
| Thanks and appreciation | You have shared a lot of your thoughts and feelings about the advisory board. Thank you very much for that, I really enjoyed listened to you. Your experiences are very valuable and interesting for us. You help us to better understand the participation of people with dementia in health care research. |
| Transparency | As part of our study, we will transcribe the interview and analyze it scientifically. Afterwards  the audio recording of our conversation will be deleted immediately. |
| Farewell | Thank you very much again, <name>! Goodbye for today and am already looking forward to our  next meeting in the advisory board. |
