# Supplementary material for: The psychological effects of research participation on people with dementia: findings from a German exploratory interview study
Source: Front Dement. 2024 Aug 7;3:1421541. doi: 10.3389/frdem.2024.1421541 (PMC11335729; doi:10.3389/frdem.2024.1421541)
Supplement: Supplementary file 2 [file Table_2.docx]

Supplementary Material

**Table 2.** Additional Results, sorted by Main category and Dimension

| **Category** | **Example statement** |
| --- | --- |
| **Emotional level** | |
| **Pride**  Self-esteem that results from one's own performance that is seen as respectful and honorable. This emotion results from the realization that one has provided an achievement worthy of recognition solely through active participation in the advisory board. The co-researchers feel proud in moments when they receive appreciative feedback on their advisory board activities. | "So, it makes me a bit proud, well, it brushes my belly a bit, as they say" (Interview 3, pos. 485)  "Well, I'm proud that I was able to help you" (Interview 2, pos. 186-187)  „So if you then realise, um, something where you say, ‚oh yes, you're right‘ then we feel good.“ (Interview 1, pos. 522)  "You repeatedly praised me so playfully" (Interview 1, pos. 278). |
| **Emotional relief**  The relief of something oppressive or burdensome. Advisory board as a place where emotional relief is possible through dialogue. | "relieving" (Interview 1, item 640)  one could "get rid of everything" (Interview 1, item 138)  caring "make[s] it easier" (Interview 1, item 368)  you could "talk things out" (Interview 4, item 42).  "Yes, it's sad too. But everyone talks about how things are going for them and what happens next" (Interview 4, pos. 170-172).  "… sad, if you had to say somewhere, um, okay, maybe there are urgent thoughts and things, no, and they're going down the drain somewhere, no. ... And everyone might have something to say.” (Interview 3, pos. 420-430) |
| **Feeling supported**  The advisory board is perceived as helpful and that the advisory board members are taken care of. | Perception in the context of the advisory board as "assistance" (Interview 1, pos. 45),  "help" (Interview 1, pos. 536)  "helpful" (Interview 1, pos. 639)  "[I] see people who take care - so they also take care of me. And I like that" (Interview 1, pos. 366-367). |
| **Security**  The feeling of being protected from potential harm in a social environment. | "CONFIDENTIAL" (Interview 1, pos. 640)  "[I] think the advisory board is also a bit of a leash, a leash that I can hold on to quite well" (Interview 3, pos. 605-606)  "[I]t is important for ME, um, um, to be within this network" (Interview 3, pos. 182) |
| **Sadness**  The feeling of sadness as a reaction to a perceived loss experienced within the context of the advisory board meetings. | "At first, um um um of course it makes me a bit sad when I realize that I can't quite follow things.“ (Interview 3, pos. 310-313)  „...it's quite good and yes, it is also sad.“ (Interview 4, pos. 170-171) |
| **Insecurity**  Loss of emotional security and self-doubt in the context of advisory board meetings | "What I sometimes REALICE myself, um, that a bit of this like, this task, that's probably part of dementia, um, the onset of dementia, um, sometimes I can't quite grasp it anymore, um, yes. ... The content, um. Which of course also makes me insecure" (Interview 3, pos. 264-271) |
| **Cognitive level** | |
| **Cognitive stimulation**  Stimulating mental processes such as concentration, memory and thinking as part of the advisory board's activities in distinction to self-help groups | "…to have an appointment for the head" (Interview 2, pos. 128)  remain "mentally moved" (Interview 2, item 135)  remain "challenged" (Interview 1, item 517)  [The advisory board is] "very, very important for the grey matter" (Interview 2, pos. 92)  [The advisory board] "brings things to light would um, which then have special significance for the further meetings and for the study itself" (Interview 3, pos. 75-77).  [through the collaboration] "certain things somehow network somewhere" (Interview 3, pos. 635) |
| **Reflection on one’s own needs**  The advisory board activity triggers reflection on one's own needs in the sense of thinking about perceived deficiencies, combined with the desire for remedy.  The reflection on needs focuses on ethical, cognitive, and behavioral aspects. | **Ethical**:  "Dignity. Yes. No assaults" (Interview 3, pos. 448)  "…to maintain sensitivity for the ... cries for help from others, um yes. Um, sensitivity for injustices, um yes. Things that don't fit somehow." (Interview 3, pos. 703-706)  **Cognitive**:  „We then learn what um Alzheimer means. And maybe we'll also learn um tips on how to make ourselves fit against major setbacks.“ (Interview 1, pos. 74-76)  „That I learn to understand my condition.“ (Interview 1, pos. 90)  "And you know what, I think I'd like to use my head more again.“ (Interview 2, pos. 49-50)“  **Behavioral**:  "… where you can talk about something like that" (Interview 1, pos. 207)  "…that everyone can bring in their own biography somewhere, um yes. ...So, what does that mean, what was my life, what did I do?" (Interview 3, pos. 687-695). |
| **Confidence**  Trust that (1) the results of their board work will be put into practice, (2) they will succeed in involving more PWD in advisory boards. (3) A confident view of their one life with dementia. | (1) „I have confidence in us.“ (Interview 1, pos. 715)  (2) „You will succeed.“ (Interview 1, pos. 792)  (3) "I know it's about forgetfulness. And I have enough evidence to prove it. Um, but this meetings with you or with ... the self-help group, um, you see, life goes on." (Interview 1, pos. 242-248). |
| **Curiosity**  The expectation of experiencing or learning something new in the future. | „I find it/ am always somewhere actually, when these appointments are, um yes in such a positive tension. Erm, what's coming today…. What will be brought to the table.“ (Interview 3, pos. 31-49)  „That's always really exciting, isn't it, what emerges/ crystallizes or what's new, isn't it?“ (Interview 3, pos. 79-81)  "But now I'm curious to see what they have to say about it [the results of work]." (Interview 2, pos. 182-183). |
| **Reflection on one's own dementia disease**  Reflection on the condition and/or progression of one's own disease is stimulated by the advisory board's work.  Differentiation between general considerations that touch on dementia as part of the self-concept and specific considerations that concern a disease-related perception of deficits within the advisory board (as subcategories). | General considerations:  „Even though I am in need myself. ... I now have the menetekel [German language; Def.: a mysterious sign of impending doom] Alzi [German language; here: Abbreviation used for Alzheimer]“ (Interview 1, pos. 732-735)  „I've now been diagnosed with Alzheimer's and, um, and it will probably get worse because I'm getting older." (Interview 1, pos. 426-428).  "I'm not quite as mentally capable as I used to be, um yes. But I think I'm still ready for it. Maybe not in two- or three-years’ time. Maybe." (Interview 3, pos. 581-585).  Disease-related perception of deficits within the advisory board:  "… always the sword of Damocles somewhere, how long [one] can still follow the content somewhere halfway." (Interview 3, pos. 183-185)  „Whereby I sometimes must think about the things that are at the advisory board and the things that are at the other meeting, um, but maybe that's also what makes it a bit like advanced Alzheimer, that I have these two meetings practically, um, or forms of meetings, um yes. Um, where I sometimes have to think, wait, where am I, where am I now, right now.“ (Interview 3, pos. 139-146)  "The task, that's probably part of the dementia, um yes, the onset of dementia, ..., sometimes you can't quite grasp that anymore...the content." (Interview 3, pos. 264-270).  „Okay, this is where I realize where I reach my limits, my intellectual limits." (Interview 3, pos. 313-316) |
| **Behavioral level** | |
| **Other activities**  Participation in the advisory board encourages further activities, e. g. in additional advisory boards. Willingness to continue to participate in working committees in the future. | „I always know that we're going out to eat [after advisory board meetings]. " (Interview 4, pos. 74-75) |
| **Being authentic**  The advisory board as a place and protected setting where co-researchers can be authentic and behave according to their own thoughts, feelings, and needs. | „Yes, you can get away with anything, you can get rid of everything.“ (Interview 1, pos. 137-138)  „And you can also make jokes.“ (Interview 1, pos. 306)  "You see, I really come out of my shell" (Interview 1, pos. 648).  „You know, and I also don't have the feeling that I'm somehow ... ashamed to talk. I'm always babbling.“ (Interview 2, pos. 272-274) |
| **Social participation**  Participation also enables the co-researchers to participate socially instead of withdrawal. | "Yes, I like going there. ... Alzheimers also leads to, um, loneliness in the end“ (Interview 1, pos. 672-674).  „I return among people, right. And that's quite a lot. Because I'm here/ with whom/ because I talk to very few people [about] something like that [dementia].“ (Interview 1, pos. 201-204)  „Don't get locked up at home, but rather join in with us ... . I did that a lot before, you know. I didn't want to go out, I didn't want to do anything.“ (Interview 2, pos. 658-670) |
